# Default “Gunel and Dickey” Bayes factors for contingency tables

**DOI:** 10.3758/s13428-016-0739-8

**Published:** 2016-06-20

**Authors:** Tahira Jamil, Alexander Ly, Richard D. Morey, Jonathon Love, Maarten Marsman, Eric-Jan Wagenmakers

**Affiliations:** 1 Department of Psychology, University of Amsterdam, Nieuwe Prinsengracht 129B, 1018 VZ Amsterdam, Netherlands; 2 School of Psychology, Cardiff University, Cardiff, UK

**Keywords:** Bayes factors, Contingency table, Sampling models, *p*-value

## Abstract

The analysis of *R*×*C* contingency tables usually features a test for independence between row and column counts. Throughout the social sciences, the adequacy of the independence hypothesis is generally evaluated by the outcome of a classical *p*-value null-hypothesis significance test. Unfortunately, however, the classical *p*-value comes with a number of well-documented drawbacks. Here we outline an alternative, Bayes factor method to quantify the evidence for and against the hypothesis of independence in *R*×*C* contingency tables. First we describe different sampling models for contingency tables and provide the corresponding default Bayes factors as originally developed by Gunel and Dickey (*Biometrika*, 61(3):545–557 (1974)). We then illustrate the properties and advantages of a Bayes factor analysis of contingency tables through simulations and practical examples. Computer code is available online and has been incorporated in the “BayesFactor” R package and the JASP program (jasp-stats.org).

Contingency tables are ubiquitous throughout psychology and the social sciences. Here we focus on the analysis of *R*×*C* contingency tables, that is, contingency tables with two categorical variables. In a contingency table, the intersection of row and column categories are known as cells. If *y*_∗∗_ is a matrix of counts with *R* rows and *C* columns then the cell entry *y*_*rc*_ corresponds to the intersection of the *r* ^th^ row with the *c* ^th^ column. For concreteness, consider Experiment 2 from the famous set of three experiments by Dutton and Aron (1974), designed to “test the notion that an attractive female is seen as more attractive by males who encounter her while they experience a strong emotion (fear) than by males not experiencing a strong emotion.” (Dutton and Aron 1974, p. 511). The experimental scenario involved a attractive female interviewer who contacted male participants to fill out a short questionnaire. To manipulate fear, a subset of participants filled out the questionnaire while balancing on a fear-arousing suspension bridge (i.e., the Capilano Canyon Suspension Bridge: five-foot wide, swaying, with low hand rails and a 230-foot drop to rocks below); the remaining subset of participants filled out the questionnaire while standing on a solid wood bridge with high handrails, positioned only 10 feet above a small stream. To measure experienced attractiveness, one of the key dependent measures was whether or not the participants later called the female interviewer (they had been given her phone number after completing the questionnaire).^1^ Table 1 summarizes the results of the study in a 2×2 contingency table with fear and attraction as categorical variables with two levels each. The hypothesis from Dutton and Aron (1974) entails that the outcomes for the two categorical variables are dependent: knowing whether a participant is in the fear-arousing condition should affect the probability that he later decides to call the interviewer.

**Table 1:** Number of men who called or did not call the female interviewer when the earlier questionnaire had been conducted on a fear-arousing suspension bridge or on a solid wood bridge

Fear	Attraction		
	Call	No call	Total
Suspension bridge	9	9	18
Solid bridge	2	14	16
Total	11	23	34

Data from Dutton and Aron (1974), Experiment 1

The top left cell entry of Table 1, *y*_11_=9, indicates that 9 men were interviewed on the suspension bridge and later called the female interviewer; the bottom left cell entry, *y*_21_=2, indicates that 2 men were interviewed on the solid bridge and later called the interviewer. In the following we use the dot notation to indicate summation; for example, Table 1 shows that a grand total of *y*_.._=34 men participated, and that of these men *y*_.1_=9+2=11 called the female interviewer, whereas *y*_.2_=9+14=23 did not. Examination of all four cell frequencies in Table 1 suggests that men were more likely to call after having been interviewed on the fear-arousing suspension bridge instead of on a solid bridge. Hence, the two categorical variables do not appear to be independent. Dutton and Aron (1974, p. 512) conclude: “In the experimental group 9 out of 18 called, in the control group 2 out of 16 called ( *χ* ^2^=5.7, *p*<.02). Taken in conjunction with the sexual imagery data, this finding suggests that subjects in the experimental group were more attracted to the interviewer.”

In order to test the hypothesis of independence in *R*×*C* contingency tables, popular methods include the *χ* ^2^ test, the likelihood ratio test, and the Fisher exact test. All these tests are classical or frequentist, and ultimately their inferential purpose rests on the interpretation of a *p*-value. The Fisherian believes this *p*-value quantifies the evidence against the null hypothesis, whereas the Neyman-Pearsonite believes it warrants the decision to reject the null hypothesis whenever *p*<*α*, with *α*=.05 as the default value (see e.g., Hubbard & Bayarri, 2003 for a discussion of the difference between the two classical paradigms). Unfortunately, all *p*-value inference is plagued by the same conceptual and practical problems (e.g., Dienes, 2011; Wagenmakers, 2007; Wagenmakers, Lee, Lodewyckx, & Iverson, 2008; Wagenmakers et al., in press; Wagenmakers et al., 2016; Wagenmakers, Morey, & Lee, in press). For example, *p*-values are sensitive to the intention with which the data were collected (i.e, they violate the Likelihood Principle, Berger & Wolpert, 1988); *p*-values cannot be used to quantify support in favor of the null-hypothesis; and finally, *p*-values are known to overestimate the evidence against the null-hypothesis (e.g., Berger & Delampady, 1987; Edwards, Lindman, & Savage, 1963). The main goal of this article is to outline an alternative, Bayes factor hypothesis test for the *R*×*C* contingency table that can be used to complement or replace the classical hypothesis tests based on *p*-values.

Bayes factors for contingency tables have a long history (e.g., Gunel & Dickey, 1974; Jeffreys, 1935, 1961; Kass & Raftery, 1995; Edwards et al., 1963). However, most of this work can be understood and used only by those with a high level of statistically sophistication, a fetish for archaic notation, and a desire for programming and debugging. At any rate, social scientists generally do not use Bayes factors for the analysis of contingency tables, and we surmise that the key reasons for this are twofold: (1) the Bayesian tests are relatively inaccessible, and (2) their practical use has not been appropriately emphasized.

The outline of this paper is as follows. The first section briefly describes four different sampling plans for contingency tables. The second section introduces the Bayes factor in general terms, and the third section gives the rationale and equations for four Bayes factors developed by Gunel and Dickey (1974) (henceforth GD74) for *R*×*C* contingency tables. The fourth section provides a simulation, and the fifth section demonstrates the application of the GD74 Bayes factors to a series of concrete examples. Following the discussion section, the Appendix provides code that illustrates how the results from the examples can be obtained from the BayesFactor package in R. The contingency table Bayes factors have also been incorporated in JASP, a free and open-source software program for statistical analyses (jasp-stats.org); see Appendix for details.

We would like to stress that our main contribution in this paper is not to propose new Bayes factors for contingency tables. Instead, our contribution was to decipher and translate the original GD74 article, implement the result in a popular software program, and demonstrate its added value by means of practical application.

## Four sampling plans

The methods developed for the Bayesian analysis of contingency tables depend on the informativeness of the design.^2^ For the case of the *R*×*C* contingency table, we follow GD74 and distinguish between the following four designs: Poisson, joint multinomial, independent multinomial, and hypergeometric. Below we consider each in turn.

### Poisson sampling scheme

Each cell count is random, and so is the grand total. Each of the cell counts is Poisson distributed. This design often occurs in purely observational work. For instance, suppose one is interested in whether cars come to a complete stop at an intersection (yes/no) as a function of the driver’s gender (male/female). When the sampling scheme is to measure all cars during one entire day, there is no restriction on any cell count, nor on the grand total.

### Joint multinomial sampling scheme

This scheme is the same as the Poisson scheme, except that the grand total (*y*_.._) is now fixed; hence, for the 2×2 table one only needs three cell counts to uniquely identify the fourth, and the cell counts are distributed as a joint multinomial. For the car example above, this scheme holds when the stopping rule is “collect data from 100 cars and then stop”.

### Independent multinomial sampling scheme

In this scheme there are two restrictions, either on the row totals or on the column totals. In other words, either all row margins or all column margins are fixed. Consequently, the cell counts are multinomially distributed within each row or column. In experimental psychology, this is the most common sampling scheme. For the car example, this scheme holds when the stopping rule is “collect data from 50 male drivers and 50 female drivers”. For the 2×2 table, two cell counts (i.e., the number of men who come to complete stop, and the number of women who come to a complete stop) suffice to uniquely identify the remaining two.

### Hypergeometric sampling scheme

In this scheme both row and column margins are fixed. For the 2×2 table, a single cell count suffices to determine the remaining three uniquely. The cell counts are said to be hypergeometrically distributed. Practical application of the hypergeometric sampling scheme is rare. For the 2×2 table, an infinite number of examples can be constructed by classifying participants according to a median split on two continuous variables. For example, suppose we have 100 participants, with income and altruism as variables of interest. The first median split creates a group of 50 rich participants and 50 poor participants; the second median split creates a group of 50 altruistic participants and 50 egotistical participants. Hence, all row and column margins are fixed, and a single cell count suffices to uniquely identify the remaining three.

GD74 devised an ingenious scheme of successive conditionalization to obtain Bayes factors for each of the four sample schemes separately. Before we describe their result the next section provides a more general outline of the Bayes factor and its advantages.

## Bayes factor basics

Denote the observed data by *y* and two competing models by $\mathcal {M}_{1}$ and $\mathcal {M}_{2}$. It follows from Bayes’ rule that the posterior model odds equals the prior model odds multiplied by the Bayes factor (Jeffreys 1961; Kass and Raftery 1995):

1 $$ \underbrace{\frac{p(\mathcal{M}_{1}\mid y)}{p(\mathcal{M}_{2} \mid y)}}_{\text{Posterior odds}} = \underbrace{\frac{p(\mathcal{M}_{1})}{p(\mathcal{M}_{2})}}_{\text{Prior odds}} \times\, \underbrace{\frac{p(y \mid \mathcal{M}_{1})}{p(y \mid \mathcal{M}_{2})}}_{\text{Bayes factor}}.  $$

Hence the Bayes factor quantifies the change from prior to posterior model odds that are brought about by the data. In this sense, the Bayes factor grades the decisiveness of the evidence that the data provide for the hypotheses under consideration (Jeffreys 1961). The Bayes factor can also be conceptualized as the ratio of marginal likelihoods of $\mathcal {M}_{1}$ versus $\mathcal {M}_{2}$ (Jeffreys 1961):

2 $$\begin{array}{@{}rcl@{}} \text{BF}_{12} &=&\frac{p(y \mid \mathcal{M}_{1})}{p(y \mid \mathcal{M}_{2})} \\ &=&\frac{{\int}_{\Theta} p(y \mid \theta, \mathcal{M}_{1})\, p(\theta \mid \mathcal{M}_{1})\, \text{d} \theta} {{\int}_{\Gamma}\, p(y \mid \gamma, \mathcal{M}_{2}) \,p(\gamma \mid \mathcal{M}_{2})\, \text{d} \gamma}. \end{array} $$

This equation shows that the relative support of the data for $\mathcal {M}_{1}$ versus $\mathcal {M}_{2}$ depends on the ratio of the prior-weighted average likelihood, that is, on the average adequacy of predictions made for data *y*. Models receive support when they provide a good account of the observed data across a relatively a large proportion of their parameter space. In contrast, highly flexible models make many predictions, and most of these will be very poor for data *y*; these poor predictions drive down the average likelihood, thereby implementing a penalty for complexity known as Occam’s razor (Myung and Pitt 1997; Lee and Wagenmakers 2013). Note that throughout this article, the first BF subscript indicates the model that is in the numerator and the second subscript indicates the model that is in the denominator; hence, BF_12_=1/BF_21_.

The framework of Bayes factors is entirely general, and applies regardless of whether $\mathcal {M}_{1}$ and $\mathcal {M}_{2}$ are nested (i.e., one is a restricted subset of the other, as is required for *p*-value null-hypothesis significance testing) or structurally different (e.g., the diffusion model versus the linear ballistic accumulator model, e.g., Donkin, Brown, Heathcote, & Wagenmakers, 2011). By fully conditioning on the observed data and by gauging strength of evidence based on predictive performance (Rouder, Morey, Verhagen, Swagman, & Wagenmakers, in press; Wagenmakers, Grünwald, & Steyvers, Wagenmakers, Morey, & Lee, 2006; in press), Bayes factors overcome several key limitations of *p*-value null-hypothesis significance testing. With Bayes factors, the null-hypothesis does not enjoy a special status and is not evaluated in isolation, but instead is always pitted against a specific alternative. Moreover, the Bayes factor provides a graded assessment of evidence and does not enforce or warrant an all or none decision in terms of “rejecting” or “failing to reject” a specific hypothesis.

In terms of interpretation, BF_12_=6.5 means that the data are 6.5 times more likely under $\mathcal {M}_{1}$ than under $\mathcal {M}_{2}$; BF_12_=0.2 means that the data are 1/0.2=5 times more likely under $\mathcal {M}_{2}$ than under $\mathcal {M}_{1}$. When we assume that the competing models are equally likely a priori (i.e., when the prior odds equal 1), the Bayes factor can be transformed to a posterior probability by dividing the Bayes factor by the Bayes factor plus 1; for example, under equal prior probability a Bayes factor of BF_12_=6.5 leads to a posterior probability for $\mathcal {M}_{1}$ of 6.5/7.5≈0.87; a Bayes factor of BF_12_=0.2 leads to a posterior probability for $\mathcal {M}_{1}$ of 0.2/1.2≈0.17.

Despite the inherently continuous nature of the Bayes factor as a measure of evidential strength, Jeffreys (1961) proposed to categorize Bayes factors in discrete categories, shown in Table 2. These categories facilitate communication and their main use is to prevent overly enthusiastic interpretation of Bayes factors in the range from 1/3−3; nevertheless, the category structure is no more than a descriptive simplification of a continuous, graded scale of evidence.^3^

**Table 2:** A descriptive classification scheme for the interpretation of Bayes factors BF_12_ (Lee and Wagenmakers 2013; adjusted from Jeffreys 1961)

Bayes factor	Posterior probability under prior equipoise	Evidence category
> 100	> 0.99	Extreme evidence for $\mathcal {M}_{1}$
30 – 100	0.97 – 0.99	Very strong evidence for $\mathcal {M}_{1}$
10 – 30	0.91 –0.97	Strong evidence for $\mathcal {M}_{1}$
3 – 10	0.75 – 0.91	Moderate evidence for $\mathcal {M}_{1}$
1 – 3	0.50 – 0.75	Anecdotal evidence for $\mathcal {M}_{1}$
1	0.50	No evidence
1/3 – 1	0.25 – 0.50	Anecdotal evidence for $\mathcal {M}_{2}$
1/10 –1/3	0.09 – 0.25	Moderate evidence for $\mathcal {M}_{2}$
1/30 – 1/10	0.03 – 0.09	Strong evidence for $\mathcal {M}_{2}$
1/100 – 1/30	0.01– 0.03	Very strong evidence for $\mathcal {M}_{2}$
< 1/100	< 0.01	Extreme evidence for $\mathcal {M}_{2}$

## Bayes factors for four sampling models

In this section we provide the GD74 Bayes factors for tests of row-column independence in contingency tables, separately for each of the four sampling schemes. All Bayes factor tests are based on a comparison of two models: one model that represents the hypothesis of row-column independence ($\mathcal {H}_{0}$) and the other model that represents the hypothesis of row-column dependence ($\mathcal {H}_{1}$). Before providing the tests in detail it is necessary to establish some notation first. Readers who are more interested in the practical application than in the statistical details are invited to skip ahead to the section with practical examples.

### Notation

Let *y*_∗∗_ be a data matrix of *R* rows and *C* columns:

3 $$ y_{**}= \left(\begin{array}{cccc} y_{11} & y_{12} & {\cdots} & y_{1C} \\ y_{21} & y_{22} & {\cdots} & y_{2C} \\ {\vdots} & {\vdots} & {\ddots} & {\vdots} \\ y_{R1} & y_{R2} & {\cdots} & y_{RC} \end{array}\right),  $$

and let *a*_∗∗_ be a matrix of prior parameters with the same dimension as the data matrix *y*_∗∗_:

4 $$ a_{**} = \left(\begin{array}{cccc} a_{11} & a_{12} & {\cdots} & a_{1C} \\ a_{21} & a_{22} & {\cdots} & a_{2C} \\ {\vdots} & {\vdots} & {\ddots} & {\vdots} \\ a_{R1} & a_{R2} & {\cdots} & a_{RC} \end{array}\right).  $$

In vector form, $\vec {y} = (y_{11}, y_{12}, . . . , y_{RC})$ and $\vec {a} = (a_{11}, a_{12}, . . . , a_{RC})$. In the following, recall that a dot is used to indicate summation across a particular dimension (row or column), and note that a star is used to indicate the entire vector of that dimension. This is clarified by the equations below:

5a $$\begin{array}{@{}rcl@{}} y_{..}&=&\sum\limits_{r} \sum\limits_{c} y_{rc}= y_{11}+y_{12}+. . . + y_{RC} \end{array} $$

5b $$\begin{array}{@{}rcl@{}} y_{*}.&=&\sum\limits_{c} y_{rc}=(y_{1.}, . . . ,y_{R.}) \end{array} $$

5c $$\begin{array}{@{}rcl@{}} y._{*}&=&\sum\limits_{r} y_{rc}=(y_{.1}, . . . ,y_{.C}) \end{array} $$

5d $$\begin{array}{@{}rcl@{}} a_{..}&=&\sum\limits_{r} \sum\limits_{c} a_{rc} \end{array} $$

5e $$\begin{array}{@{}rcl@{}} a_{*}.&=&\sum\limits_{c} a_{rc}=(a_{1.}, . . . ,a_{R.}) \end{array} $$

5f $$\begin{array}{@{}rcl@{}} a._{*}&=&\sum\limits_{r} a_{rc}=(a_{.1}, . . . ,a_{.C}) \end{array} $$

5g $$\begin{array}{@{}rcl@{}} \xi_{*}.&=&a_{*}.-(C-1) \end{array} $$

5h $$\begin{array}{@{}rcl@{}} \xi ._{*}&=&a ._{*}-(R-1) \end{array} $$

5i $$\begin{array}{@{}rcl@{}} \xi_{..} &=&a_{..}-(R-1)(C-1) \end{array} $$

5j $$\begin{array}{@{}rcl@{}} \mathcal{D}(a_{**})& =&\prod \frac{\Gamma(a_{rc})} {\Gamma(a_{..})}. \end{array} $$

For the matrix of prior parameters *a*_∗∗_ (i.e., the gamma shape parameters of the Poisson rates for the cell counts, see below), a default value is obtained when each *a*_*rc*_=*a*=1 – in the multinomial case, this indicates that every combination of parameter values is equally likely a priori. Higher values of *a* bring the predictions of $\mathcal {H}_{1}$ closer to those of $\mathcal {H}_{0}$; the prior distribution under *a*=10, for instance, may be thought of as an uninformative *a*=1 prior distribution that has been updated using 9 hypothetical observations in each cell of the table. For the data in Table 1, *y*_.._=34, *y*_∗_.=(18,16) a vector of row totals, and *y*._∗_=(11,23) a vector of column totals. When *a*=1 then *a*_∗_.=(2,2) and *a*._∗_=(2,2). Consequently, *ξ*_∗_. is a vector of ones of length *R*, the number of rows, *ξ*._∗_ is a vector of ones of length *C*, the number of columns, and *ξ*_.._=3 . Finally, $\mathcal {D()}$ is a Dirichlet function defined in Eq. 5j (Albert 2007; Gunel and Dickey 1974).

### Four Bayes factors

Below we describe, separately for the four sampling schemes, the GD74 contingency table Bayes factors in support of the row-column independence model $\mathcal {H}_{0}$ over the row-column dependence model $\mathcal {H}_{1}$. Bayes factors are often difficult to calculate, as they are obtained by integrating over the entire parameter space, a process that is non-trivial when the integrals are high-dimensional and intractable. GD74’s Bayes factors, however, only require computation of common functions such as gamma functions, for which numerical approximations are readily available. GD74 achieved this simplicity through a series of model restrictions and data conditionalization.

In order to describe how GD74 simplified their Bayes factor calculations, we must first introduce the idea of a “conditional” Bayes factor. Consider testing a simple normal mean and variance with two participants. The specific hypotheses do not matter; we instead focus on the information in the data. If we were sampling sequentially, we might compute the Bayes factor for our hypothesis after the first participant, and then after the second participant. The second Bayes factor takes into account all the data, and includes all the information from both participants. We can also look at the Bayes factor due to having observed participant 2’s data, already taking into account the data from participant 1. This Bayes factor represents the “extra” information about the hypothesis offered by participant 2 over and above that offered by participant 1. We can call it the Bayes factor for participant 2 given, or conditional on, participant 1. However, we can partition the data in other ways besides participants. Since the sample mean and variance jointly capture all the information in the data, we can also describe the Bayes factor for the sample mean conditioned on knowing the sample variance.

In the context of contingency tables, there are logical ways of partitioning the data. To begin, we partition the data into a part that contains the information about the overall quantity of observations, and a part that contains the information about how cells differ from one another. To compute the evidence assuming that the total number of observations is fixed, we look at the change from the Bayes factor using only the first part of the data (the total number of observations) to the Bayes factor conditioned on the whole data set. Due to the way GD74 parameterized their models –model parameters corresponding to the components of the partition– this successive conditionalization produces Bayes factors that are easy to compute.

1. Bayes factor under the Poisson sampling scheme
  Under this sampling scheme, none of the cell counts are fixed. Each cell count is assumed to be Poisson distributed: *y*_*rc*_∼Poisson(*λ*_*rc*_). Each of the rate parameters *λ*_*rc*_ is assigned a conjugate gamma prior with shape parameter *a* and scale parameter *b*: *λ*_*rc*_∼Γ(*a*_*rc*_,*b*). Here, ${\Gamma }(a_{rc},b)=\frac {b^{a}}{\Gamma (a)} \lambda ^{a-1} e^{-b\lambda }$, *λ*>0, *a*>0 and *b*>0 and Γ(*a*) is the gamma function Γ(*a*)=(*a*−1)!. The Bayes factor for independence under the Poisson sampling scheme is (Equation 4.2 in GD74):
  6 $$\begin{array}{@{}rcl@{}} \text{BF}^{P}_{01} &=& (1+1/b)^{(R-1)(C-1)} \frac{\Gamma(y_{..}+\xi_{..} )} {\Gamma(\xi_{..} )}\\ &&{\prod}_{rc}\frac{\Gamma(a_{rc})}{\Gamma(y_{rc}+a_{rc})} \frac{\mathcal{D}(y_{*}.\!+\xi_{*}.)}{\mathcal{D}(\xi_{*}.)} \frac{\mathcal{D}(y._{*}\!+\xi ._{*})}{\mathcal{D}(\xi ._{*})} \end{array} $$
  where *b*=*R*×*C*×*a*/*y*_.._ is the default value of the gamma scale parameter suggested by GD74.^4^
  For the 2×2 table with *a*=1, the Bayes factor simplifies to
  7 $$\label {Poisson1} \text{BF}^{P}_{10} = \frac {8\, (y_{..}+1)(y_{1.}+1)}{(y{..}+4)(y{..}+2)}\left[\frac {y_{11}! \,y_{12}!\, y_{21}!\, y_{22}!\, y{..}!}{(y_{1.}+1)! \,y_{2.}!\,y_{.1}!\,y_{.2}!}\right].  $$
2. Bayes factor under the joint multinomial sampling scheme
  Under this sampling scheme, the grand total *y*_.._ is fixed. Cell counts are assumed to be jointly multinomially distributed: (*y*_11_,...,*y*_*rc*_)∼Multinomial(*y*_.._,*π*_∗∗_). The prior distribution on the multinomial parameters is the conjugate Dirichlet distribution: *π*_∗∗_∼Dirichlet(*a*_∗∗_). The Bayes factor for independence under the joint multinomial sampling scheme is (Equation 4.4 in GD74; see also O’Hagan, Forster, & Kendall, 2004, p. 351 and Albert,^2007^, p. 178):
  8 $$ \text{BF}^{M}_{01} = \frac{\mathcal{D}(y_{*}.+\xi_{*}.)}{\mathcal{D}(\xi_{*}.)} \frac{\mathcal{D}(y._{*}+\xi ._{*})} {\mathcal{D}(\xi ._{*})} \frac{ \mathcal{D}(a_{**})} {\mathcal{D}(y_{**}+a_{**})}.  $$
  For the 2×2 table with *a*=1, the Bayes factor simplifies to
  9 $$\label {Multinomial1} \text{BF}^{M}_{10} = \frac {6\, (y_{..}+1)(y_{1.}+1)}{(y{..}+3)(y{..}+2)}\left[\frac {y_{11}! \,y_{12}!\, y_{21}!\, y_{22}!\, y{..}!}{(y_{1.}+1)! \,y_{2.}!\,y_{.1}!\,y_{.2}!}\right].  $$
3. Bayes factor under the independent multinomial sampling scheme
  Under this sampling scheme, one margin (rows or columns) in the contingency table is fixed. Cell counts are assumed to be independently multinomially distributed. The Bayes factor for independence under this sampling scheme is (Equation 4.7 in GD74):
  10 $$ \text{BF}^{I}_{01} = \frac{\mathcal{D}(y._{*}+\xi ._{*})}{\mathcal{D}(\xi ._{*})} \frac{\mathcal{D}(y_{*}.+a_{*}.)} {\mathcal{D}(a_{*}.)} \frac {\mathcal{D}(a_{**})}{\mathcal{D}(y_{**}+a_{**})}.   $$
  This Bayes factor is derived under the assumption that the row margins are fixed. To derive the Bayes factor under the assumption that the column margins are fixed, it suffices to interchange the rows and columns in Eq. 10.
  11 $$ \text{BF}^{I}_{01} = \frac{\mathcal{D}(y_{*}.+\xi_{*}.)}{\mathcal{D}(\xi_{*}.)} \frac{\mathcal{D}(y._{*}+a._{*})} {\mathcal{D}(a._{*})} \frac {\mathcal{D}(a_{**})}{\mathcal{D}(y_{**}+a_{**})}.   $$
  For the 2×2 contingency table, the Bayes factor for the independent multinomial sampling plan reduces to a test for the equality of two proportions, *𝜃*_1_ and *𝜃*_2_. Under the default setting *a*=1, Eq. (11) then simplifies to (de Bragança Pereira & Stern, 1999; Jeffreys, 1935; Wagenmakers, Lodewyckx, Kuriyal, & Grasman, 2010):
  12 $$ \text{BF}^{I}_{01} = \frac{\left(\begin{array}{c}y_{.1}\\y_{11}\end{array}\right) \left(\begin{array}{c}y_{.2}\\y_{12}\end{array}\right)} {\left(\begin{array}{c}y_{.1}+y_{.2}\\y_{11}+y_{12}\end{array}\right)} \frac{(y_{.1}+1)(y_{.2}+1)}{(y_{.1}+y_{.2}+1)},  $$
  where the left-hand side features binomial coefficients.
  The Bayes factor $\text {BF}^{I}_{01}$ –or its inverse, which quantifies the evidence for $\mathcal {H}_{1}$, that is, $\text {BF}^{I}_{10} = 1/\text {BF}^{I}_{01}$– is a two-sided test. In experimental disciplines, however, researchers often have strong prior beliefs about the direction of the effect under scrutiny. For instance, Dutton and Aron (1974) set out to test whether emotional arousal stimulates attraction, not whether emotional arousal dampens attraction. A one-sided Bayes factor that respects the directional nature of the alternative hypothesis needs to assess the support for hypothesis $\mathcal {H}_{+}: \theta _{1} > \theta _{2}$ or $ \mathcal {H}_{-}:\theta _{1} < \theta _{2}$. These one-sided Bayes factors can be obtained easily (Morey & Wagenmakers, 2014; Pericchi, Liu, & Torres, 2008). To see this, we first decompose the desired one-sided Bayes factor, say BF_+0_, into two parts:^5^
  13 $$\begin{array}{@{}rcl@{}} \text{BF}_{+0} &=&\frac{ p(y \mid \mathcal{H}_{+})}{ p(y \mid \mathcal{H}_{0})}\\ &=&\frac{ p(y \mid \mathcal{H}_{+})}{ p(y \mid \mathcal{H}_{1})}\times \frac { p(y \mid \mathcal{H}_{1})} {p(y \mid \mathcal{H}_{0})}\\ &=&\text{BF}_{+1} \times \text{BF}_{10}. \end{array} $$
  Thus, in order to obtain the one-sided BF_+0_, we need to adjust the two-sided BF_10_ by the factor BF_+1_, which quantifies the evidence for the directional alternative hypothesis $\mathcal {H}_{+}$ over the undirectional alternative hypothesis $\mathcal {H}_{1}$. To obtain this evidence, we use a simple procedure outlined by Klugkist, Laudy, and Hoijtink (2005), who noted that BF_+1_ equals the ratio of posterior and prior mass under $\mathcal {H}_{1}$ that is consistent with the restriction postulated by $\mathcal {H}_{+}$. That is, $\text {BF}_{+1} = p(\theta _{1}>\theta _{2} \mid y, \mathcal {H}_{1}) / p(\theta _{1}>\theta _{2} \mid \mathcal {H}_{1})$; for symmetric prior distributions, the correction factor further simplifies to $\text {BF}_{+1} = 2 \times p(\theta _{1}>\theta _{2} \mid y, \mathcal {H}_{1})$. From this expression it is evident that incorporating the direction of the effect in the specification of the alternative hypothesis can increase the Bayes factor in its favor by no more than twofold.
4. Bayes factor under the hypergeometric sample scheme.
  In a 2×2 table the conditional distribution of *y*_11_ given both margins fixed (i.e., *p*(*y*_11_∣*y*_1._,*y*_2._,*y*_.1_,*ψ*)) is a noncentral hypergeometric distribution:
  14 $$ p(y_{11} \mid y_{1.},y_{2.},y_{.1}, \psi) \,=\, \frac{ \left(\begin{array}{c}y_{.1}\\y_{11}\end{array}\right) \left(\begin{array}{c}y_{.2}\\y_{1.}-y_{11}\end{array}\right) \psi^{y_{11}}} {\sum\nolimits_{i=\max(0, y_{1.}-y_{.2})}^{\min(y_{1.},y_{.1})} \left(\begin{array}{c}y_{.1}\\ i\end{array}\right) \left(\begin{array}{c}y_{.2}\\y_{1.}-i\end{array}\right) \psi^{i}}  $$
  for 0<*y*_1._≤*y*_.1_+*y*_.2_ and $ \max (0, y_{1.}-y_{.2}) \leq y_{11} \leq \min (y_{1.},y_{.1})$. The noncentral hypergeometric distribution equals the hypergeometric distribution when the odds ratio (*ψ*)=1.
  The Bayes factor for independence under the hypergeometric sampling scheme is (Equation 4.11 in GD74):
  15 $$ \text{BF}^{H}_{01} = \frac{\mathcal{D}(a_{**})\sum g(y_{**}; y_{..},a_{**})} {\mathcal{D}(y_{**}+a_{**})\left(\begin{array}{c}y_{..}\\y_{*}.\end{array}\right) \left(\begin{array}{c}y_{..}\\y._{*}\end{array}\right)},  $$
  where
  16 $$ g(y_{**}; y_{..},a_{**}) = \left(\begin{array}{c}y_{..}\\y_{**}\end{array}\right) \frac{\mathcal{D}(y_{**}+a_{**})} {\mathcal{D}(a_{**})},  $$
  and $\sum $ is a summation over $y_{**}^{\prime }$ with all margins fixed.
  For the 2×2 table with *a*=1, Eq. 15 is equivalent to the Bayes factor proposed by Jeffreys (1961, p. 264):
  17 $$ \text{BF}^{H}_{10} = \frac{y_{11}! \,y_{12}!\, y_{21}!\, y_{22}!\, y{..}!} {(y_{1.}+1)! \,y_{2.}!\,y_{.1}!\,y_{.2}!},   $$
  where *y*_1._=min(*y*_1._,*y*_2._,*y*_.1_,*y*_.2_), that is, the smallest of the four marginal totals.

For all four Bayes factors, the parameter matrix *a*_∗∗_ quantifies the prior uncertainty. By default, each element of the matrix is assigned the same number *a*. For the Dirichlet distribution, the priors are uniform across their range when *a*=1. This is the default choice of GD74 and we will explore the Bayes factors outlined here with this choice in mind. As usual, robustness of statistical conclusions may be checked by varying the prior precision along a plausible range of values. Note the uniform choice assumes that differences between marginal probabilities are expected to be large. If smaller effects are expected, the *a* parameter may be increased.

### Relation between the four Bayes factors for the 2×2 table

To quantify the relationship between the Bayes factors for each of the four sampling plans discussed above we focus on the 2×2 contingency table and use the default prior setting *a*=1. It is then possible to derive the ratio for pairs of Bayes factors; for instance, the ratio between the Bayes factor for the Poisson sampling plan and the hypergeometric sampling plan is obtained as $\text {BF}^{P}_{10}/\text {BF}^{H}_{10}$. All ratios of Bayes factors are shown in Table 3; the cell in the first row and final column shows the outcome for $\text {BF}^{P}_{10}/\text {BF}^{H}_{10}$.

**Table 3:** Ratios of default Bayes factors for 2×2 contingency tables under the four different sampling plans

	$\text {BF}^{M}_{10}$	$\text {BF}^{I}_{10}$	$\text {BF}^{H}_{10}$
$\text {BF}^{P}_{10}$	$\frac {4(y_{..}+3)}{3(y{..}+4)}$	$\frac {8 (y_{1.}+1)(y_{2.}+1)} {(y{..}+4)(y{..}+2)}$	$\frac {8(y_{..}+1)(y_{1.}+1)} {(y{..}+4)(y{..}+2)}$
$\text {BF}^{M}_{10}$		$\frac {6 (y{1.}+1)(y_{2.}+1)} {(y_{..}+3) (y_{..}+2)}$	$\frac {6 (y{..}+1)(y_{1.}+1)}{(y_{..}+3) (y_{..}+2)}$
$\text {BF}^{I}_{10}$			$\frac { (y{..}+1)}{(y_{2.}+1)}$

The ratios are obtained by dividing the Bayes factor shown in rows by that shown in columns. Note that BF_10_=1/BF_01_. See text for details

Table 3 reveals that the evidence in favor of the row-column dependence hypothesis $\mathcal {H}_{1}$ decreases with the successive conditioning on the table margins and totals. In other words, the Bayes factor BF_10_ is largest for the Poisson sampling plan, and smallest for the hypergeometric sampling plan.

## Simulation

To explore the behavior of the four Bayes factors further we conducted two simulations, each with synthetic data from a 2×2 contingency table. In the first simulation, we took the table $\vec {y}=(3, 3, 2, 5)$ with *y*_.._=3+3+2+5=13 as a point of departure, with an log odds ratio of 0.91 and a corresponding 95 % confidence interval of ( −1.37,3.20). We then created a total of 30 contingency tables by multiplying each cell count by a factor *c*, where *c*=1,2,...,30. Hence, the grand total number of observations varied from *y*_.._=13 at *c*=1, through *y*_.._=195 at *c*=15, to *y*_.._=390 at *c*=30.

**Fig. 1:**
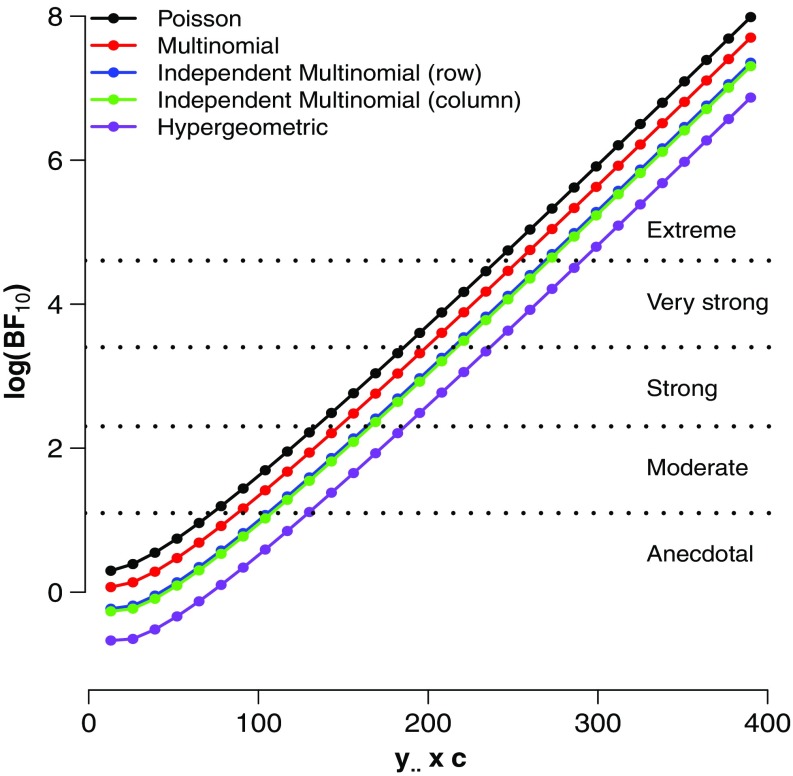
Four GD74 Bayes factors for different enlargement factors (c) of the $\vec {y}=(3, 3, 2, 5)$ table. See text for details

For each of the 30 contingency tables, we calculated the GD74 Bayes factors under each of the four sampling schemes. Figure 1 shows the results. As expected, the evidence against the null hypothesis increases with sample size. For low sample sizes, the Bayes factors indicate that the evidence is merely anecdotal, favoring $\mathcal {H}_{1}$ —or sometimes even $\mathcal {H}_{0}$— by less than a factor of three. In addition, the different Bayes factors all show a linear increase in the log Bayes factor as sample size increases. The evidential gap between the Bayes factor for the Poisson sampling scheme and the hypergeometric sampling scheme approximately spans an entire Jeffreys category. For instance, when *c*=10 the hypergeometric $\text {BF}^{H}_{10} = 3.04$, whereas the Poisson $\text {BF}^{P}_{10} = 9.19$, suggesting that the differences between the Bayes factors under the different sampling models can be substantial.

**Fig. 2:**
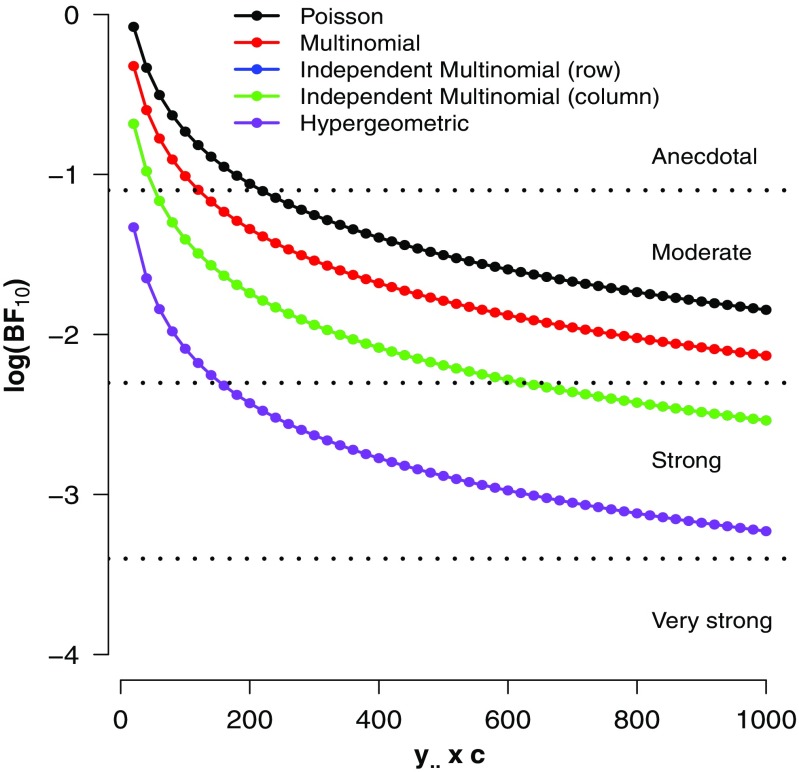
Four GD74 Bayes factors for different enlargement factors (c) of the $\vec {y}=(5, 5, 5, 5)$ table. See text for details

In the second simulation, we took the table $\vec {y}=(5, 5, 5, 5)$ as a point of departure, with an odds ratio of 1. We created a total of 50 contingency tables by multiplying each cell count by a factor *c*, where *c*=1,2,...,50. Hence, the grand total number of observations varied from *y*_.._=20 at *c*=1, through *y*_.._=500 at *c*=25, to *y*_.._=1000 at *c*=50. For each table, the counts are uniformly distributed across the cells and this should yield the maximum possible evidence for $\mathcal {H}_{0}$. As before, for each of the 30 contingency tables we calculated the GD74 Bayes factors under each of the four sampling schemes. Figure 2 shows the results.

As expected, the evidence in favor of $\mathcal {H}_{0}$ –independence between rows and columns– increases with sample size. The speed of the increase is less pronounced than it was in the first simulation — a reflection of the general rule that for nested models, it is often relatively difficult to find compelling evidence in favor of the absence of an effect (Jeffreys 1961). Consistent with the mathematical relation displayed in Table 3, the evidential order has reversed; the strongest evidence is now provided by the hypergeometric Bayes factor, whereas the Poisson Bayes factor is the most reluctant of the four in its support for $\mathcal {H}_{0}$. The order-reversal suggests that, with default GD74 priors, the Poisson model has more prior mass in the vicinity of the null hypothesis than does the hypergeometric model.

In sum, the simulations confirm that the Bayes factor support grows with sample size; they also highlight that differences between the four Bayes factors cannot easily be ignored, not even asymptotically.

## Examples

This section underscores the practical relevance of the GD74 Bayes factors by discussing a concrete example for each of the four sampling plans. For comparison, we also report the results from *p*-value null-hypothesis statistical testing.

### Poisson sampling example: fathers and sons

Table 4 shows the contingency table for professional occupations of 775 fathers and their sons; the data were collected by Miss Emily Perrin and published by Pearson (1904, p. 33). The diagonal entries –shown in bold italic– indicate the number of cases where the son’s occupation matches that of his father.

**Table 4:** The occupation of fathers and their sons. Data reported in (Pearson 1904, p. 33)

Father’s occupation	Son’s occupation													
	1	2	3	4	5	6	7	8	9	10	11	12	13	14
1	***28***	0	4	0	0	0	1	3	3	0	3	1	5	2
2	2	***51***	1	1	2	0	0	1	2	0	0	0	1	1
3	6	5	***7***	0	9	1	3	6	4	2	1	1	2	7
4	0	12	0	***6***	5	0	0	1	7	1	2	0	0	10
5	5	5	2	1	***54***	0	0	6	9	4	12	3	1	13
6	0	2	3	0	3	***0***	0	1	4	1	4	2	1	5
7	17	1	4	0	14	0	***6***	11	4	1	3	3	17	7
8	3	5	6	0	6	0	2	***18***	13	1	1	1	8	5
9	0	1	1	0	4	0	0	1	***4***	0	2	1	1	4
10	12	16	4	1	15	0	0	5	13	***11***	6	1	7	15
11	0	4	2	0	1	0	0	0	3	0	***20***	0	5	6
12	1	3	1	0	0	0	1	0	1	1	1	***6***	2	1
13	5	0	2	0	3	0	1	8	1	2	2	3	***23***	1
14	5	3	0	2	6	0	1	3	1	0	0	1	1	***9***

Labels: 1-army, 2-art, 3-teacher, clerk, civil servant, 4-crafts, 5-divinity, 6-agriculture, 7-landownership, 8-law, 9-literature, 10-commerce, 11-medicine, 12-navy, 13-politics and court, 14-scholarship and science

For illustrative purposes, we assume that sampling was based on a Poisson scheme, such that any cell count can take on any value, and the grand total was not fixed in advance. A frequentist test of independence between rows and columns yields $\chi ^{2}_{(df=169, y_{..}=775)}=1005.45$ and *p*<.001: we can reject the the null hypothesis of independence and conclude that there is an association between the profession of fathers and their sons. However, the *p*-value does not quantify how much these data should shift our belief. To address this question we calculate the Poisson GD74 Bayes factor and obtain $\log {\text {BF}_{10}^{P}} = 262.21$, indicating extreme evidence for the hypothesis that there exists an association between the occupations of fathers and their sons.

### Joint multinomial sampling example: job satisfaction

**Fig. 3:**
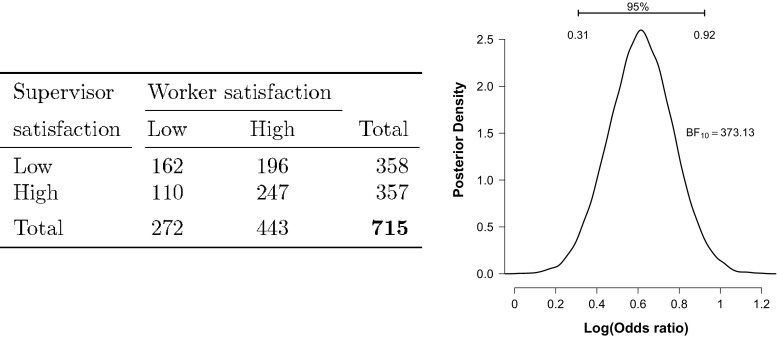
Data from a 1968 job satisfaction questionnaire among 715 blue collar industrial workers in Denmark (Andersen 1990). Left panel: contingency table; right panel: posterior distribution of the log odds ratio

The left panel of Fig. 3 shows data from a 1968 job satisfaction questionnaire among 715 blue collar industrial workers in Denmark (Andersen 1990). One expects an association between supervisor satisfaction and worker satisfaction, expressed by an abundance of counts on the diagonal cell entries.

For illustrative purposes, we assume that sampling was based on a joint multinomial scheme, such that the grand total of 715 workers was fixed. A frequentist test of independence between rows and columns yields $\chi ^{2}_{(df=1, y_{..}=715)}=15.81$ and *p*<.001: we can reject the the null hypothesis of independence and conclude that there is an association between the satisfaction of supervisors and workers. However, the *p*-value does not quantify how much these data should shift our belief. To address this question we calculate the joint multinomial GD74 Bayes factor and obtain $\text {BF}_{10}^{M} = 373.13$, indicating extreme evidence for the hypothesis that there exists an association between the satisfaction level of supervisors and workers.

In addition, the right panel of Fig. 3 shows the posterior distribution of the log odds ratio (as can be obtained using JAGS, Plummer, 2003, or the BayesFactor package, Morey & Rouder, 2015; see Appendix for code). The 95 % credible interval for the log odds ratio spans the range from 0.31 to 0.92, and the median value equals $\log (1.85) = 0.61$; note that independence corresponds to a log odds ratio of zero. The classical estimate of the log odds ratio is 0.62 and the classical 95 % confidence interval is (0.31,0.92).

### Independent multinomial example: dolls

**Fig. 4:**
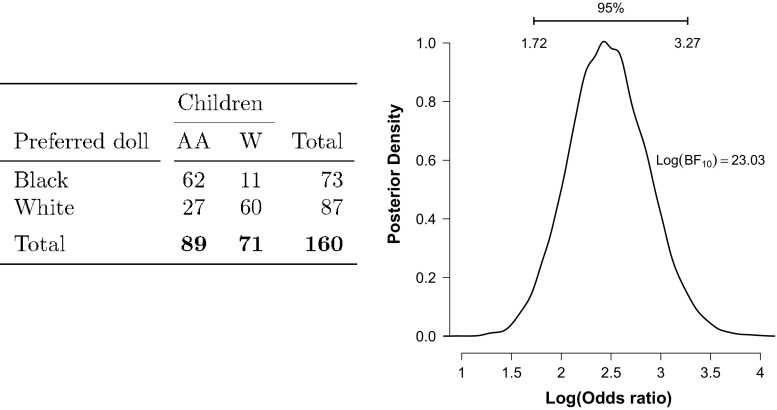
Racial preference among Nebraska school children in 1969. Data from (Hraba and Grant 1970). Left panel: contingency table; AA=African American; W=White. Right panel: posterior distribution of the log odds ratio

The left panel of Fig. 4 shows data from a classic study on racial preference among school children (Hraba and Grant 1970). Among 160 Nebraska children aged 4-8, 62 out of 89 African American children (69 %) preferred to play with a black doll instead of a white doll, whereas 60 out of 71 white children (84 %) preferred to play with a white doll instead of a black doll.

For illustrative purposes, we assume that sampling was based on an independent multinomial scheme, such that the crucial test involves a comparison of two proportions. A frequentist test of independence between rows and columns yields $\chi ^{2}_{(df=1, y_{..}=160)}=46.71$ and *p*<.001: we can reject the the null hypothesis of independence and conclude that there is an association between children’s race and the color of the doll they prefer to play with. However, the *p*-value does not quantify how much these data should shift our belief. To address this question we calculate the independent multinomial GD74 Bayes factor and obtain $\log \text {BF}_{10}^{I} = 23.03$, indicating strong evidence for the hypothesis that there exists an association between children’s race and the color of the doll they preferred to play with.

In addition, the right panel of Fig. 4 shows the posterior distribution of the log odds ratio. The 95 % credible interval for the log odds ratio spans the range from 1.73 to 3.26, and the median value equals log(11.82) = 2.47. The classical estimate of the log odds ratio is 2.52 and the classical 95 % confidence interval is (1.74,3.31).

### Hypergeometric example: siblings

The left panel of Table 5 shows data from 30 first-born children, aged 3-5, and their attitude towards a soon-to-be-born sibling (Kramer & Gottman, 1992; as reported in Anderson, 1993, p. 14–15). The contingency table has been constructed using a median split for the variables age (younger versus older) and sibling acceptance (lower versus higher). Hence, both margins are fixed and the sampling scheme is hypergeometric. At issue is the question of whether there exists a relation between age and sibling acceptance.

**Table 5:** Sibling acceptance data from Kramer and Gottman (1992) as reported in Anderson (1993, p. 14–15)

Age	Sibling acceptance		Total
	Lower	Higher	
Younger	9	6	**15**
Older	6	9	**15**
Total	**15**	**15**	**30**

A frequentist test of independence between rows and columns yields $\chi ^{2}_{(df=1, y_{..}=30)}= 1.2$ and *p*=0.27: we fail to reject the null hypothesis of independence and conclude that there is insufficient evidence for an association between age and sibling acceptance. However, the *p*-value does not quantify how much these data should shift our belief in favor of the independence hypothesis. To address this question we calculate the hypergeometric GD74 Bayes factor and obtain $\text {BF}_{10}^{H} = 0.39$, indicating that the observed data are about 1/0.39=2.56 times more likely under the null hypothesis of independence than under the alternative hypothesis of dependence.

## Concluding comments

In this article, we discussed a series of default Bayes factors for the analysis of *R*×*C* contingency tables and we illustrated their use with concrete examples. Following Gunel and Dickey (1974), we distinguished four sampling schemes. In order of increasing restriction, these are Poisson, joint multinomial, independent multinomial, and hypergeometric. The prior distributions for each model are obtained by successive conditioning on fixed cell frequencies or margins.

The use of Bayes factors affords researchers several concrete advantages. For instance, Bayes factors can quantify evidence in favor of the null hypothesis and Bayes factors may be monitored as the data accumulate, without the need for any kind of correction (e.g., Rouder, 2014). The latter advantage is particularly pronounced when the relevant data are obtained from a natural process that unfolds over time without any predefined stopping point.

It may be argued that these Bayesian advantages have long been within reach, as Bayes factors for contingency tables have been developed and proposed well over half a century ago (Jeffreys 1935). Nevertheless, for the analysis of contingency tables researchers almost exclusively use classical methods, obtaining *p*-values through chi-square test and likelihood ratio tests. One reason for the neglect of Bayesian methods in the empirical sciences is that they lack implementation in user-friendly software packages. We have tried to overcome this obstacle by providing R syntax (see Appendix) and by incorporating the GD1974 Bayes factor in the BayesFactor package through the function contingencyTableBF(). In addition, we have also made the GD74 Bayes factors available in the open-source statistical package JASP (www.jasp-stats.org).

Before closing, let us return to the data in Table 1. The classical analysis suggested that men who were interviewed on the fear-arousing bridge rather than the solid wood bridge called the female interviewer more often ( *p*<.02). The relevant GD74 Bayes factor assumes an independent multinomial sampling scheme; in the case of the 2 table, the test simplifies to a comparison between two proportions. The Bayes factor yields $\text {BF}_{10}^{I}=5.31$, which indicates that data are about 5 times more likely under $\mathcal {H}_{1} $ than they are under $\mathcal {H}_{0}$. However, the authors’ hypothesis implies that the alternative hypothesis is one-sided. Following the method described above and elsewhere (e.g., Morey & Wagenmakers, 2014), we compute the Bayes factor for $\mathcal {H}_{+}$ versus $\mathcal {H}_{0}$ to be *B* *F*_+0_=10.50; according to the classification scheme proposed by Jeffreys, this is strong but not overwhelming evidence for the presence of an effect.

The GD74 Bayes factors are but one of many Bayesian analyses that have been proposed for the analysis of *R*×*C* contingency tables. Other early approaches include Altham (1969, 1971); Good (1965, 1967); Good and Crook (1987); Jeffreys (1935, 1961). The approach by Altham focuses on parameter estimation rather than on hypothesis testing, whereas the approaches advocated by Good and by Jeffreys are similar to those outlined here. Another alternative Bayesian approach is Poisson regression or log-linear modeling (e.g., Forster, 2010; Overstall & King, 2014), a discussion of which is beyond the scope of the current work. Also note that the GD74 approach hinges on the use of prior distributions of a particular form; if the user wishes to specify prior distributions from a different family, analytical results may no longer be possible, and one would have to turn to Markov chain Monte Carlo techniques (e.g., Gilks, Richardson, & Spiegelhalter, 1996; Gamerman & Lopes, 2006).

In closing, we believe that the GD74 Bayes factors allow an additional and valuable perspective on the analysis of *R*×*C* contingency tables. By making these Bayes factors available in several software packages, researchers should feel uninhibited to make use of the methodology and, at a minimum, confirm that their conclusions are robust to the statistical paradigm that is used to analyze the data.

## Figures and Tables

**Fig. 5:**
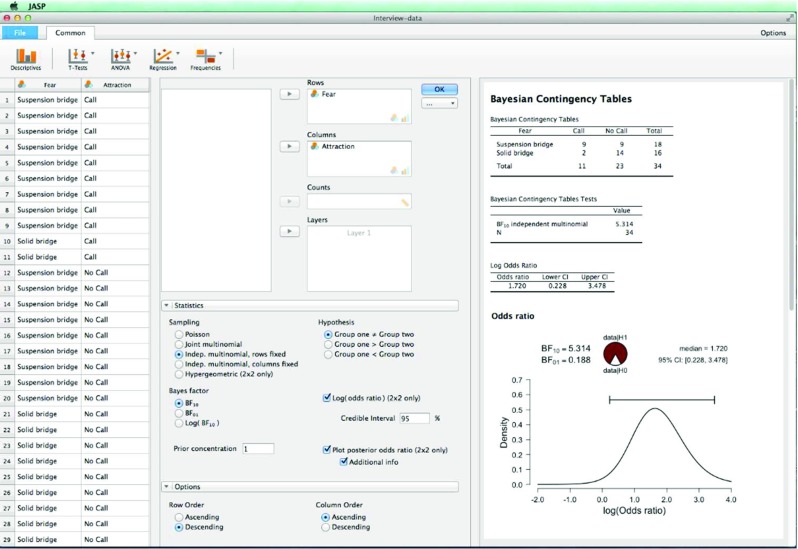
A screenshot of JASP for the analysis of a contingency table

## Appendix A: Bayes Factor R Code

This appendix provides the R code that produces the Bayes factors for the examples discussed in the main text. The main function, contingencyTableBF(), has been incorporated in the R “BayesFactor” package (Morey and Rouder 2015).

The syntax for the contingencyTableBF() function has the following form:

contingencyTableBF(x, sampleType,

fixedMargin = NULL, priorConcentration = 1,

...)

where the argument x is an “R” by “C” data matrix and sample type specifies the sampling plan ( “poisson”, “jointMulti”, “indepMulti”, “hypergeom”). The argument fixedMargin is for the independent multinomial sampling plan, to fix one of the margins (“rows” or “cols”); finally, the argument priorConcentration allows the user to deviate from the default prior setting where *a*=1.

### A.1 Poisson sampling scheme

Below is the Bayes factor R code and the resulting output for the analysis of the data shown in Table 4 (occupations of fathers and their sons):

> BFP_10 <- contingencyTableBF(data,

sampleType = "poisson",

        priorConcentration = 1)

> BFP_10 Bayes factor analysis

--------------

[1] Non-indep. (a=1) : 4.11512e+115

Against denominator:

    Null, independence, a = 1

---

Bayes factor type: BFcontingencyTable,

poisson

# The log (Bayes factor)

> BFP_10@bayesFactor$bf

[1] 266.212

### A.2 Joint multinomial sampling scheme

Below is the Bayes factor R code and the resulting output for the analysis of the data shown in the left panel of Fig. 3 (job satisfaction):

> data<- matrix(c(162, 110, 196, 247), 2, 2)

> BFM_10 <- contingencyTableBF(data,

sampleType = "jointMulti",

   priorConcentration = 1)

> BFM_10

Bayes factor analysis

--------------

[1] Non-indep. (a=1) : 373.134

Against denominator:

    Null, independence, a = 1

---

Bayes factor type: BFcontingencyTable,

joint multinomial

> BFM_10@bayesFactor$bf [1] 5.921938

Note that the last number that is returned is the log of the Bayes factor, such that exponentiating it provides the Bayes factor (i.e., $\exp {5.921938} = 373.134$).

### A.3 Independent multinomial sampling scheme

Below is the Bayes factor R code and the resulting output for the analysis of the data shown in the left panel of Fig. 4 (dolls and race):

> data=matrix(c(62,27,11,60),c(2,2))

>

> BFI_10 <- contingencyTableBF(data,

sampleType = "indepMulti",

fixedMargin = "cols",

priorConcentration = 1)

> BFI_10

Bayes factor analysis

--------------

[1] Non-indep. (a=1) : 10029550038

Against denominator:

Null, independence, a = 1

---

Bayes factor type: BFcontingencyTable,

independent multinomial

> BFI_10@bayesFactor$bf

[1] 23.03

Here we also provide the Bayes factor R code and the resulting output for the analysis of the data shown in Table 1 (arousal and attraction):

> data=matrix(c(9, 2, 9, 14), c(2, 2))

> BF <- contingencyTableBF(data,

sampleType = "indepMulti",

fixedMargin = "rows",

priorConcentration = 1)

> BF

Bayes factor analysis

--------------

[1] Non-indep. (a=1) : 5.313538

Against denominator:

    Null, independence, a = 1

---

Bayes factor type: BFcontingencyTable,

independent multinomial

> BF@bayesFactor$bf

[1] 1.670258

### A.4 Hypergeometric sampling scheme

Below is the Bayes factor R code and the resulting output for the analysis of the data shown in Table 5 (sibling acceptance):

> data=matrix(c(9, 6, 6, 9), c(2, 2))

> BFH_10 <- contingencyTableBF(data,

sampleType = "hypergeom",

priorConcentration = 1)

> BFH_10

Bayes factor analysis

--------------

[1] Non-indep. (a=1) : 0.3870194

Against denominator:

    Null, independence, a = 1

---

Bayes factor type: BFcontingencyTable,

hypergeometric

> BFH_10@bayesFactor$bf

[1] -0.9492805

This Bayes factor is implemented for 2×2 contingency table only.

## Appendix B: JASP

The GD74 Bayes factors are also available in JASP, a free and open-source statistical software package that will be familiar to users of SPSS (jasp-stats.org). In JASP, the user drags and drops variables to input boxes, and subsequently selects analysis options through mouse clicks. A screenshot of JASP is provided in Fig. 5.

## Appendix C: JAGS Code

The JAGS code below implements the joint multinomial sampling model and computes the log odds ratio.

# Multinomial Sampling model

model{

        for ( i in 1:rc) {

             alpha[i ]<- 1

        }

        theta[1:rc] ˜ ddirch(alpha[1:rc])

        y[1:rc] ˜ dmulti(theta[1:rc],n)

        log_OR <- log((theta[1]*theta[4])/

        (theta[2]*theta[3]))

}

The R code below runs the JAGS model and plots the posterior distribution of the log odds ratio.

rm(list=ls())

y <- c(162,110,196,247)

n=sum(y)

rc=4 data <-list("y", "n","rc") # to be

passed on to JAGS

myinits <- list( list(alpha=c(1,1,1,1)))

# parameters to be monitored:

parameters <- c("theta", "log_OR")

samples <- jags(data, inits=NULL,

parameters,

        model.file ="Multinomial_Model.txt",

        n.chains = 1, n.iter = 20000,

        n.burnin = 1, n.thin = 1, DIC = T)

samples.mcmc <- as.mcmc(samples)

densityplot(samples.mcmc)

logOR<- samples$BUGSoutput$sims.list$log_OR

par(cex.main = 1.5,

mar = c(5, 6, 4, 5) + 0.1,

mgp = c(3.5, 1, 0), cex.lab = 1.5,

font.lab = 2, cex.axis = 1.3,

bty = "n", las=1)

digitsize<-1.2

z<-density(logOR)

x.mode <- z$x[i.mode <- which.max(z$y)]

setEPS()

postscript("Plot_Jobs.eps")

y.mode <- z$y[i.mode]

lim<-max(z$x)-min(z$x)

ylim0 <- c(0,1.1*y.mode )

xlow<-unname(stats::quantile(logOR,

p =0.0001))

xhigh<-unname(stats::quantile(logOR,

p =0.9999))

xticks <- pretty(c(xlow,xhigh), min.n= 3)

plot(z$x,z$y,type="l", lty=1, lwd=2,

xlim=range(xticks), ylim=ylim0,

        axes=F,

        main=" ", xlab="Log(Odds ratio) ",

        ylab="Posterior Density")

axis(1, line=0.3, at=xticks, lab=xticks)

axis(2)

# plot 95% confidence interval

x0 <- quantile(logOR,p=c(.025,.975))[[1]]

x1 <- quantile(logOR,p=c(.025,.975))[[2]]

arrows(x0, 1.07*y.mode, x1, 1.07*y.mode,

length = 0.05, angle = 90, code = 3, lwd=2)

text(1, 1.5, expression(’BF’[10] == 713.8),

cex=digitsize)

text(0.6, 2.85, "95%" ,cex=digitsize)

text(x0, 2.55, round(x0, digits = 2)

,cex=digitsize)

text(x1, 2.55, round(x1, digits = 2)

, cex = digitsize)

# quartz.save("Plot_Jobs.eps", type="pdf")

dev.off()

## Footnotes

1
^1^ Not all participants accepted the phone number. The analysis here focuses only on those participants that accepted the number.

2
^2^ In classical statistics also, different tests exist for the separate sampling plans (e.g., compare the Fisher exact test to the Barnard exact test, Barnard, 1945). When the sample size is large the differences become negligible.

3
^3^ The authors are divided on the merits of Jeffreys’ classification scheme. Author RDM notes that the scheme introduces information into the analysis that is not justified by Bayesian theory itself; what is “strong” evidence is an extra-Bayesian consideration, and there is no reason that 10 should be the criterion for “strong” evidence in all, or even in most. As (Kass and Raftery 1995) note, assessments of the strength of evidence will often be contextual, and at any rate, probability theory itself provides the interpretation of the Bayes factor as the change in model odds. The Bayes factor needs no further interpretation. Other authors of this manuscript, however, believe that Jeffreys’ scheme can serve as a helpful guide.

4
^4^ Note that for the other sampling schemes the *b* parameter plays no role.

5
^5^ As before, the first BF subscript indicates the model in the numerator, and the second subscript indicates the model in the denominator of Eq. 2; hence, BF_+0_=1/BF_0+_.
